# Comparison of definitions of coronary artery reference sizes and effects on stent selection and evaluation of stent expansion

**DOI:** 10.1007/s10554-023-02890-2

**Published:** 2023-07-05

**Authors:** Lene Nyhus Andreasen, Evald Høj Christiansen, Lone Juul Hune Mogensen, Niels Ramsing Holm

**Affiliations:** 1 Department of Cardiology, Aarhus University Hospital, Aarhus, Denmark; 2 Aarhus University Hospital, Palle Juul-Jensens Boulevard 99, Aarhus, 8200 Denmark

**Keywords:** Coronary reference estimations, Optical coherence tomography, Intervascular imaging, Stent expansion

## Abstract

Accurate determination of coronary reference size is essential for optimal stent selection and evaluation of stent expansion during percutaneous coronary intervention (PCI). Several approaches for reference size estimation have been published with no universal agreement. The aim of this study was to investigate if potential differences in coronary reference size estimation lead to differences in stent and balloon selection and in detection of stent under expansion. Definitions for coronary reference size estimation, stent size selection, and stent expansion were identified in 17 randomized controlled trials. The identified methods were applied in a population of 32 clinical cases. Reference size estimates ranged up to 1.35mm, and indicated nominal stent size ranged up to 1.0 mm in the same case depending on method. Mean relative stent expansion ranged from 54±12% to mean 100±29% depending on the applied reference method. Choice of method for reference size estimation using intravascular imaging may influence stent selection and greatly affects evaluation of post-PCI stent expansion.

**Supplementary Information:**

The online version contains supplementary material available at 10.1007/s10554-023-02890-2.

## Introduction

Estimation of coronary artery reference sizes is crucial for planning and evaluation of percutaneous coronary intervention (PCI). At present, most procedures are guided by visual estimation of the reference vessel size. This method suffers from angiographic ambiguity, lack of vessel wall information, out of plane magnification, and is highly operator dependent [1].

Intravascular ultrasound (IVUS) and optical coherence tomography (OCT) are increasingly used for procedural guiding during PCI [2]. Both imaging modalities allow for detailed and precise evaluation of coronary artery dimensions [2, 3]. Coronary atherosclerosis is frequently shown by IVUS and OCT to be more extensive than it appears by angiography and hence, determination of the reference size may differ if assessed by IVUS and OCT compared with angiography [4, 5].

Accurate determination of reference size is crucial for optimal stent selection and in verification of stent expansion which is strongly correlated to clinical outcome [6, 7]. Definitions for imaging guided stent selection and optimal stent expansion are not firmly established and differ substantially between guidelines, consensus papers and several study designs [3, 8–11]. The aim of this study was to evaluate if different methods for coronary reference size estimation exist and if potential differences could lead to differences in stent size selection and in evaluation of stent expansion.

## Methods

This study comprised (1) identification of published reference methods, and (2) comparison of identified methods in a clinical dataset.

### Published reference methods, stent size selection and definitions of stent expansion

A systematic search was performed in Medline to identify published randomized control trials (RCT) using drug eluting stent (DES) and either (1) IVUS-guided PCI vs. angiography-guided PCI, (2) IVUS-guided PCI vs. OCT-guided PCI, (3) IVUS-guided PCI vs. OCT-guided PCI vs. angio-guided PCI, or (4) OCT-guided PCI vs. angiography-guided PCI.

Methods for measuring coronary reference size, stent size selection, and optimal stent expansion criteria were extracted from identified studies.

### Analyzed population

The identified methods for reference estimations, stent size selection and expansion calculation were applied and evaluated in a population of clinical cases. A total of 32 cases with pre-PCI and post-PCI OCT from the DOCTOR Fusion study and the SORT OUT VII OCT sub-study were available for analysis [12, 13]. DOCTOR Fusion was a prospective, observational study evaluating an early application for co-registration of OCT scans and coronary angiography [12]. SORT OUT VII OCT was a randomized, comparison of two different drug-eluting-stents in patients with stable coronary artery disease or acute coronary syndrome [13]. Both studies were approved by The Central Denmark Region Committees on Health Research Ethics and all patients provided written informed consent.

### Optical coherence tomography (OCT)

OCT recordings were acquired using the Abbott OCT system (Ilumien or Ilumien OPTIS) after intracoronary administration of nitroglycerin. Pullback speed was either 18 mm/sec or 36 mm/sec and pullback length was 54 or 75 mm. Corresponding OCT recordings acquired pre and post-PCI were matched using stent edges and side branches (SB) as landmarks. All cases were analyzed offline using the Aptivue offline OCT analysis system (Abbott, US).

### Comparison of reference size methods

Pre-PCI and post-PCI OCT recordings were matched on frame level. All identified reference estimation methods were performed with measurements distal and proximal to the matched pre-PCI stent segment and post-PCI stent segment.

Reference size estimations were excluded if (1) the stent edge was located at a large SB take-off (diameter ≥ 2 mm), (2) the edge was not visible due to unflushed blood, or (3) the edge was not visible due to the guiding catheter positioned inside the proximal end of the stent. All analyses were performed by the same highly experienced observer[13, 14].

### Comparison of methods for evaluation of stent expansion

**Fig. 1:**
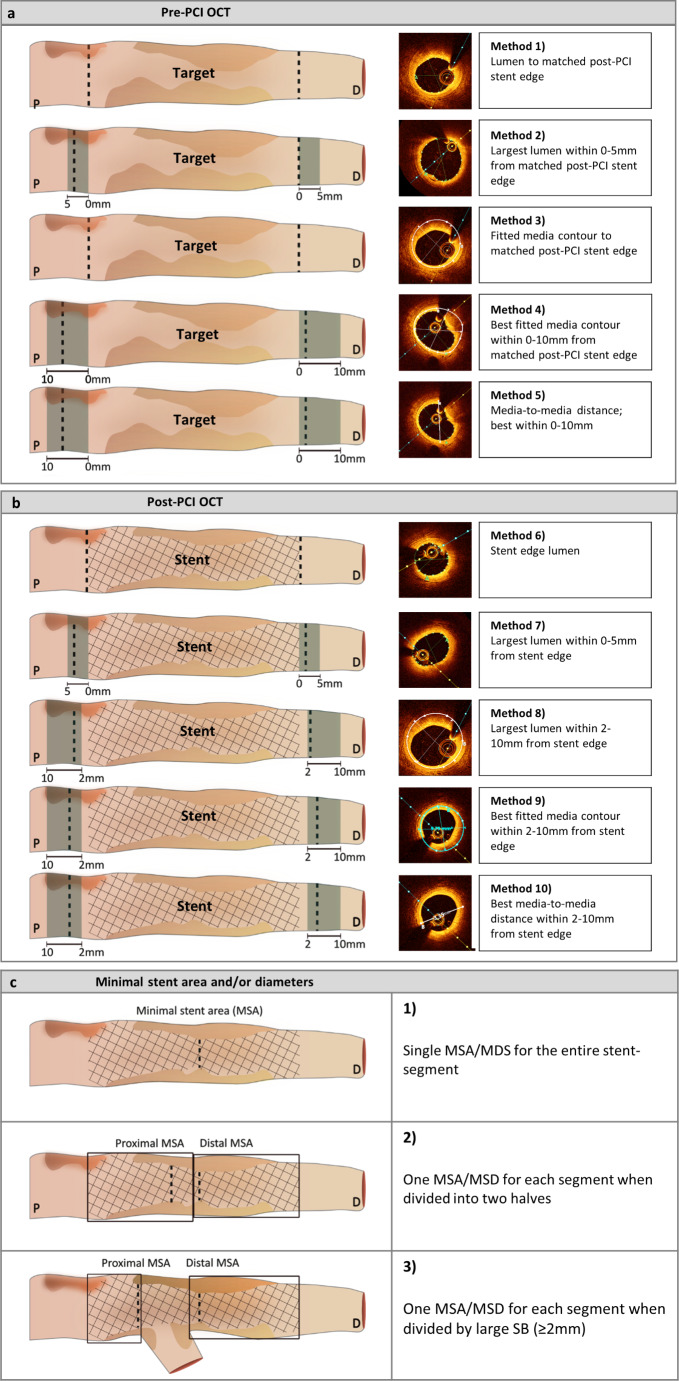
Position and methods for different reference estimations and minimal stent area (MSA) and/or minimal stent diameter (MSD). **a:** References obtained in pre-PCI OCT-runs. **b:** References obtained in post-PCI OCT-runs. **c:** Classification of different MSA and/or MSD obtained in each clinical case. *OCT*: Optical coherence tomography. *PCI*: Percutaneous coronary intervention

Identified definitions for optimal stent expansion were applied in analysis of the 32 clinical cases. Minimal stent area (MSA) and minimal stent diameter (MSD) were measured in a segmental fashion as either (1) a single measurement of MSA and MSD for the entire stent-segment, (2) MSA and MSD for each segment when the stent-segment was divided into two equal halves, or (3) MSA and MSD for each segment when the stent-segment was divided by a large SB (≥ 2 mm) branch point (Fig. 1c).

For calculation of stent expansion the following reference values were used, either (1) the distal reference alone, or (2) the mean of the distal – and proximal references, and in turn from either (1) pre-PCI reference estimations, or (2) post-PCI reference size estimations. The relative expansion value was calculated as: $100 x \frac{Minimal stent area \left(MSA\right)}{Reference area}$ or: $100 x \frac{Minimal stent diameter \left(MSD\right)}{Rederence diameter}$ .

### Statistics

Continuous variables are presented as mean ± standard deviation (normal distribution) or median + interquartile range (IQR) (non-normal distribution). Categorical variables are shown as absolute number and percentages. Bland-Altman plots including 95% limits of agreement was used to illustrate and assess the agreement and variation between different methods. One-way repeated measures ANOVA was performed to determine if there were differences between means for reference size and stent expansion methods. Maychly’s test was used to test if the assumption of sphericity, i.e. the variances of the differences between all combinations of related groups must be equal, was violated. In case of violation, the Greenhouse-Geisser correction was used. In selected cases, two methods were compared using Student’s paired t-test or Wilcoxon test as appropriate according to distribution of data. Statistical analyses were performed using STATA version 17 (Stata Inc. College Station, TX).

## Results

A total of 17 RCTs were identified; 9 studies comparing IVUS-guided with angiography-guided PCI [15–23], 2 studies comparing IVUS-guided with OCT-guided PCI [10, 24], a single study comparing IVUS-guided and OCT-guided with angio-guided PCI [8], and 5 studies comparing OCT-guided PCI with angiography-guided PCI [2, 9, 25–27]. Of these, two studies are ongoing (OCTOBER [9] and ILUMIEN IV [27] (Table 1, supplementary table).

**Table 1:** Overview of different stent selection criteria

	Distal measurement only			Distal or proximal measurement		Mean of distal and proximal		Unspecific			
	Distal ref. size (study specific down and up-sizing)	Distal media (ratio 0:8)	Distal lumen (ratio 1:1)	Distal OR prox. (the smallest) EEL, if EEL ≥ 180°	Distal OR prox. lumen (the smallest) if EEL visible < 180°	Distal + prox. EEL	Distal + prox. Lumen	Stent/vessel diameter 0.8:1	Ref. Vessel diameter	NA	QCA
HOME DES IVUS, 2010										x	
Habara et al. 2013							x				x *
The AVIO Trial, 2013										x	
CTO-IVUS, 2015										x	
AIR-CTO, 2015								x			
IVUS XPL, 2015										x	
OCTACS, 2015											x
RESET, 2015										x	
Tan et al. 2015										x	
Zhang et al. 2016											x
ILUMIEN III, 2016				x	x						
DOCTORS, 2016									x		
OCT STEMI, 2017											x
OPINION, 2017						x	x				
ULTIMATE, 2018		x	x								
ILUMIEN IV Trial	x										
OCTOBER Trial	x										

*second line, NA: Not available, QCA: Quantitative coronary angiography

## Coronary reference size

A total of 12 different reference size definitions were identified in 17 clinical studies (Table 2, supplementary table).

**Table 2:** Overview on “optimal stent expansion” and “cut-off values”

	NA	Absolute value	MSA to distal pre lumen area	MSA to distal post lumen area	MSA to distal lumen area (pre/post unknown)	Respective MSA to respective post lumen area	Respective MLD to respective pre media-reference	MSA to mean pre lumen area	MSA to mean post lumen area	MSA to mean lumen area (pre/post unknown)	MSA to ref. area (distal/prox. & pre/post unknown)	Specific study criteria	MSA ≥ 95%	MSA ≥ 90%	MSA > 80%	MSA > 70%	MSA > distal reference	NA
HOME DES IVUS, 2010		x		x										x				
Habara et al. 2013			x											x		x		
The AVIO trial, 2013												x					x	
CTO-IVUS, 2015					x												x	
AIR-CTO, 2015											x				x			
IVUS-XPL, 2015					x												x	
OCTACS, 2015									x									
RESET, 2013											x							x
Tan et al., 2015								x						x				
Zhang et al., 2016	x																	x
ILUMIEN III, 2016						x							x	x				
DOCTORS 2016											x				x			
OCT STEMI, 2017			x					x						x	x			
OPINION, 2017										x				x				
ULTIMATE, 2018		x			x									x				
ILUMIEN IV Trial						x								x				
OCTOBER Trial							x							x				

*MSA*: minimal stent area. *NA*: Not available

### Stent selection criteria

An overview of identified stent selection criteria are shown in Table 1.

### Optimal stent expansion criteria

Table 2 presents an overview of the definitions for “optimal expansion” defined in the 17 clinical studies

### Comparison of reference methods

A total of 10 different reference methods were applied in the 32 study cases (Fig. 1a-b). The distal, proximal and mean reference values are presented in Table 3.

**Table 3:** Reference estimations using different positions and methods and results of minimal stent expansion calculations

Table 3	Distal reference			Proximal reference			Mean (average distal + proximal)		
Pre-PCI reference estimations	N	Diameter (mm)		N	Diameter (mm)		N	Diameter (mm)	
1) Corresponding lumen	31	2.91 ± 0.77		26	2.99 ± 0.66		25	2.93 ± 0.64	
2) Largest lumen within 0-5 mm	31	3.05 ± 0.73		26	3.32 ± 0.70		25	3.14 ± 0.65	
3) Corresponding fitted media circle	30	3.58 ± 0.83		20	3.82 ± 0.68		19	3.68 ± 0.71	
4) Best fitted media-circle 0-10 mm	31	3.59 ± 0.80		25	4.01 ± 0.70		24	3.75 ± 0.68	
5) Best media-media 0-10 mm	31	3.52 ± 0.79		26	4.04 ± 0.74		25	3.73 ± 0.69	
One-way repeated ANOVA		*P* < 0.005			*P* < 0.005			*P* < 0.005	
Post-PCI reference estimation									
6) Stent edge	32	3.01 ± 0.68		29	3.23 ± 0.52		29	3.12 ± 0.56	
7) Largest lumen within 0-5 mm	32	3.11 ± 0.65		29	3.45 ± 0.65		29	3.28 ± 0.61	
8) Largest lumen within 2-10 mm	30	2.97 ± 0.61		20	3.56 ± 0.75		19	3.35 ± 0.65	
9) Best fitted media-circle 2-10 mm	30	3.59 ± 0.79		20	4.34 ± 0.76		19	4.01 ± 0.73	
10) Best media-media 2-10 mm	30	3.50 ± 0.74		20	4.26 ± 0.76		19	3.93 ± 0.71	
One-way repeated ANOVA		*P* < 0.005			*P* < 0.005			*P* < 0.005	
	Distal part			Proximal part			One single stent segment		
Minimal stent lumen	N	Minimal stent diameter	Minimal stent area	N	Minimal stent diameter	Minimal stent area	N	Minimal stent diameter	Minimal stent area
Separated in halves	32	2.86 ± 0.49	6.64 ± 2.24	31	2.95 ± 0.55	7.10 ± 2.69	32	2.81 ± 0.50	6.31 ± 2.31
Separated by SB > 2 mm	15	2.88 ± 0.50	6.74 ± 2.20	12	3.05 ± 0.46	7.51 ± 2.32	-	-	-

Reference estimations using different positions and methods and results of minimal stent expansion calculations. P-values are results from the one-way ANOVA analyze. *MSA*: Minimal stent area. *MSD*: Minimal stent diameter. *PCI*: Percutaneous coronary intervention. *SB*: Side branch

### Pre PCI reference size methods

**Fig. 2:**
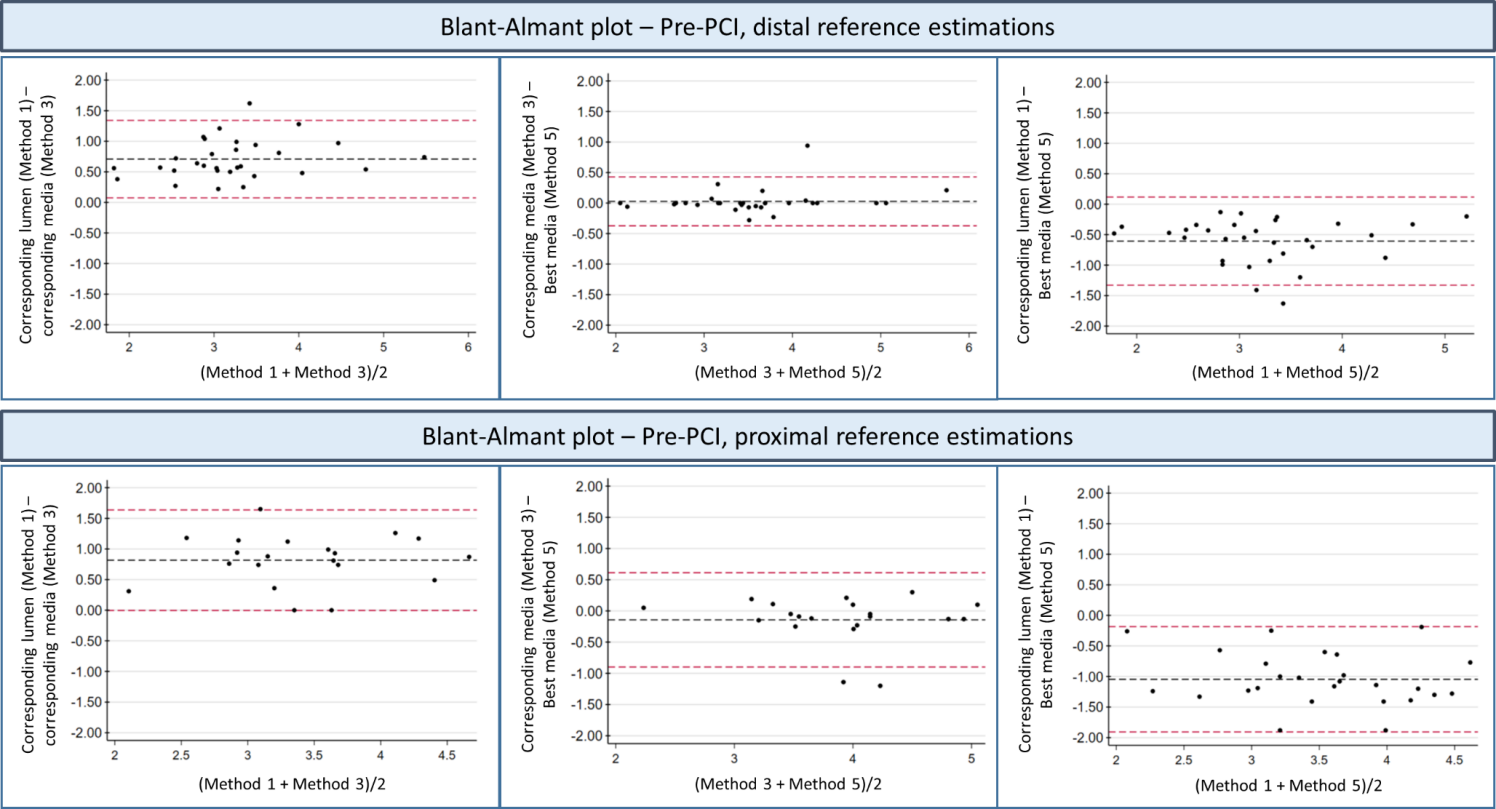
Bland-altman plot for selected pre-PCI references. Method 1 and 3: measured at the same vessel position (same frame) but different methods (Method 1: Lumen estimation, Method 3: Media-media estimation). Method 3 and 5: media-media estimation but two different positions (Method 3: corresponding stent edge position. Method 5: best media-media within 0-10 mm from corresponding stent edge. Method 1 and 5: different measurement and different positions

Smallest diameter reference size was achieved by the pre-PCI distal stent edge lumen (Method 1) (2.91 mm ± 0.77 mm), and the largest diameter reference size was derived by the pre-PCI proximal media-to-media distance (Method 5) (4.04 mm ± 0.74 mm). One-way repeated measures ANOVA for means of the references methods performed at the distal position, at the proximal position and a mean of two all showed statistical significant differences between the 5 methods (distal p < 0.001, proximal: p < 0.001, mean: p < 0.001). Selected Bland-Altman plots with key comparison of methods are presented in Fig. 2 (method 1 and 3 (references measured at the same frame position), method 3 and 5 (both references are media-media estimations but at different reference position) and method 1 and 5 (different reference estimation and position).

### Post-PCI evaluation of reference size methods

Reference size estimates after stenting (post-PCI) ranged according to method from 2.97 mm ± 0.61 mm (distal, largest lumen within 2-10 mm from stent edge (Method 8)) to 4.34 mm ± 0.76 mm (proximal best fitted media contour 2-10 mm from stent edge (Method 9)). Results from the one-way repeated measures ANOVA analysis for the post-PCI distal references, the post-PCI proximal references and the mean of the post-PCI distal and proximal references all showed statistical significant differences between methods ( p < 0.001, p < 0.001, p < 0.001). When measuring the reference size within a distance 0-10 mm from the stent edge, the largest lumen was found just at the stent edge in 47% (distal) and 41% (proximal) of analyzed cases. Bland-Altman plot for selected references methods are shown in supplementary Fig. 1).

### Comparison of reference size estimated pre - and post PCI

Pre-PCI edge lumen size (Method 1) and pre-PCI media contour size (Method 3) were measured at the same vessel position as the post-PCI edge lumen size (Method 6). The reference diameter size pre-PCI was larger for the media based estimations: distal: ∆0.71 mm ± 0.33 mm, p < 0.05 (n = 30), proximal: ∆0.81 mm ± 0.42 mm, p < 0.05 (n = 20), mean: ∆0.76 mm ± 0.28 mm, p < 0.05 (n = 25). The differences of the two lumen diameter size estimations (pre-PCI matched stent edge (Method 1) and post-PCI stent edge (Method 6) were: distal ∆0.08 mm ± 0.29 mm, p = 0.13 (n = 31), proximal: ∆0.23 mm ± 0.29, p < 0.05 (n = 26), and mean: ∆0.17 ± 0.19, p < 0.05 (n = 25). Post-PCI stent edge was larger than pre-PCI matched stent edge in all three references positions (Table 3).

### Post-PCI evaluation of stent expansion by a single MSA/MSD measurement

**Fig. 3:**
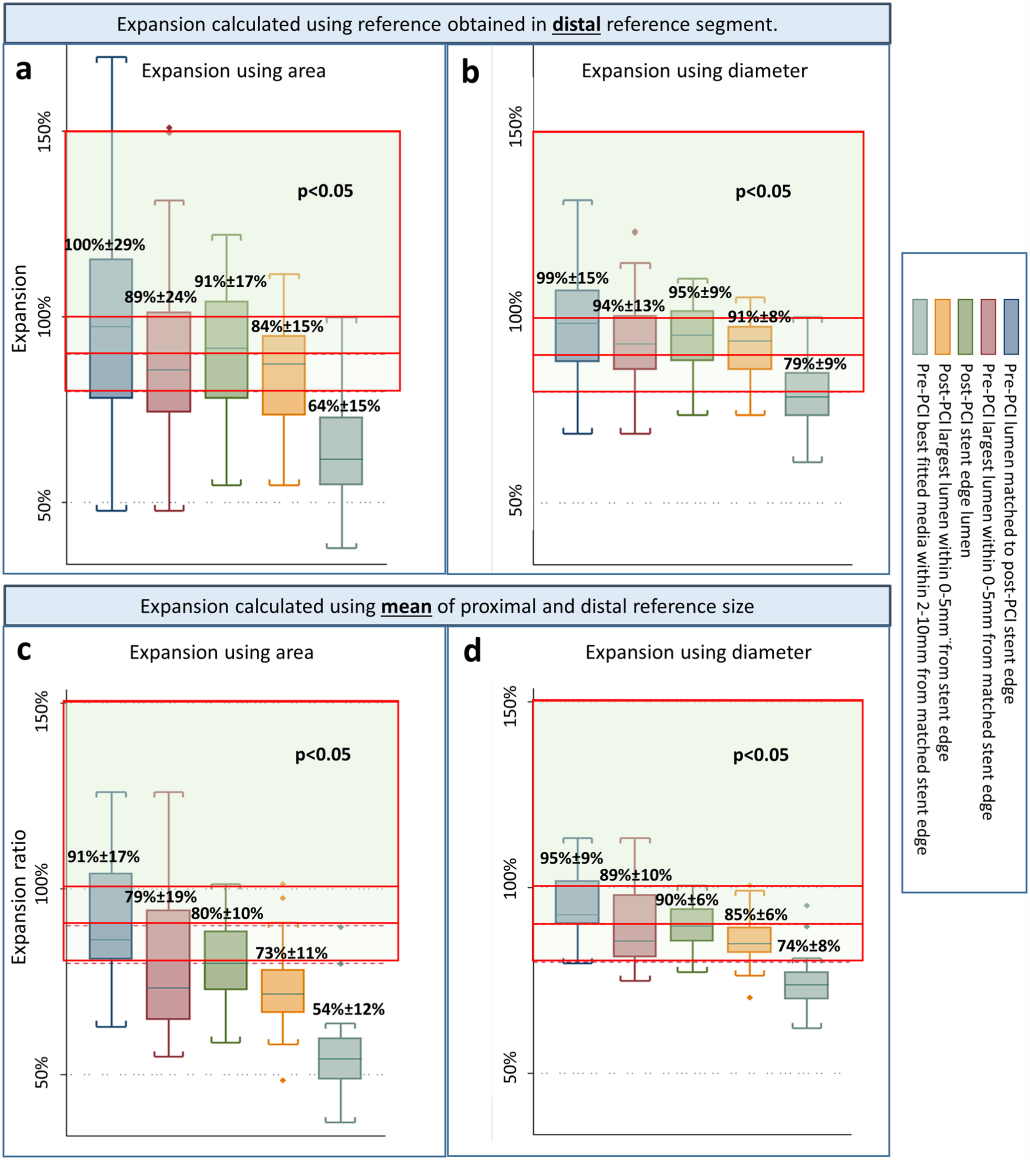
Expansion calculations using the stented segment as one single segment and **(a)** the distal reference, or **(b)** the mean of proximal and distal reference for calculation. Box-plot indicates median + IQR. Numbers placed above boxes are means ± SD. P-values are results from one-way repeated ANOVA. Red lines separate expansion by either 80%, 90% or 100%. *PCI*: Percutaneous coronary intervention

Figure 3 show box-plots of the mean expansion calculated by a single MSA – or MSD measurement from the entire stented segment and either (1) a distal reference estimation (Figs. 2b and 3a) or (2) the mean reference estimation (Figs. 2d and 3c). Expansion results are presented using 5 selected reference methods: (1) pre-PCI matched stent edge lumen (Fig. 1a1, Fig. 3 blue boxes), (2) pre-PCI largest lumen within 0-5 mm from stent edge (Fig. 1a2, Fig. 3 purple boxes), (3) post-PCI stent edge lumen (Fig. 1b6, Fig. 3 green boxes), (4) post-PCI largest lumen within 0-5 mm from stent edge (Fig. 1b7, Fig. 3 orange boxes), and (5) pre-PCI best fitted media contour within 0-10 mm from matched stent edge (Fig. 1a4, Fig. 3 grey boxes).

The largest relative expansion was shown by evaluating MSA in relation to a reference size obtained as a lumen area at the distal pre-PCI stent edge position (mean: 100% ± 29%) (Fig. 3a, blue box). The smallest relative expansion was shown using MSA and the mean of the proximal and the distal reference measured from the pre-PCI OCT-run as best fitted media contour within 0–10 mm from stent edge (mean: 54%±12%) (Fig. 3c, grey box). Results of the one-way ANOVA analysis all showed statistically significant difference between methods (p < 0.05).

Area based estimation of expansion in percent (Fig. 3a and 3c), indicated lower degree of expansion than diameter-based assessment in the same cases (Fig. 3b and d). The largest difference was found in analysis of the pre-PCI media contour (area-based expansion: 54% ±12% (Fig. 3c, grey box), diameter-based expansion: 74%±8%) (Fig. 3d, grey box).

### Segmental post-PCI evaluation of stent expansion

**Fig. 4:**
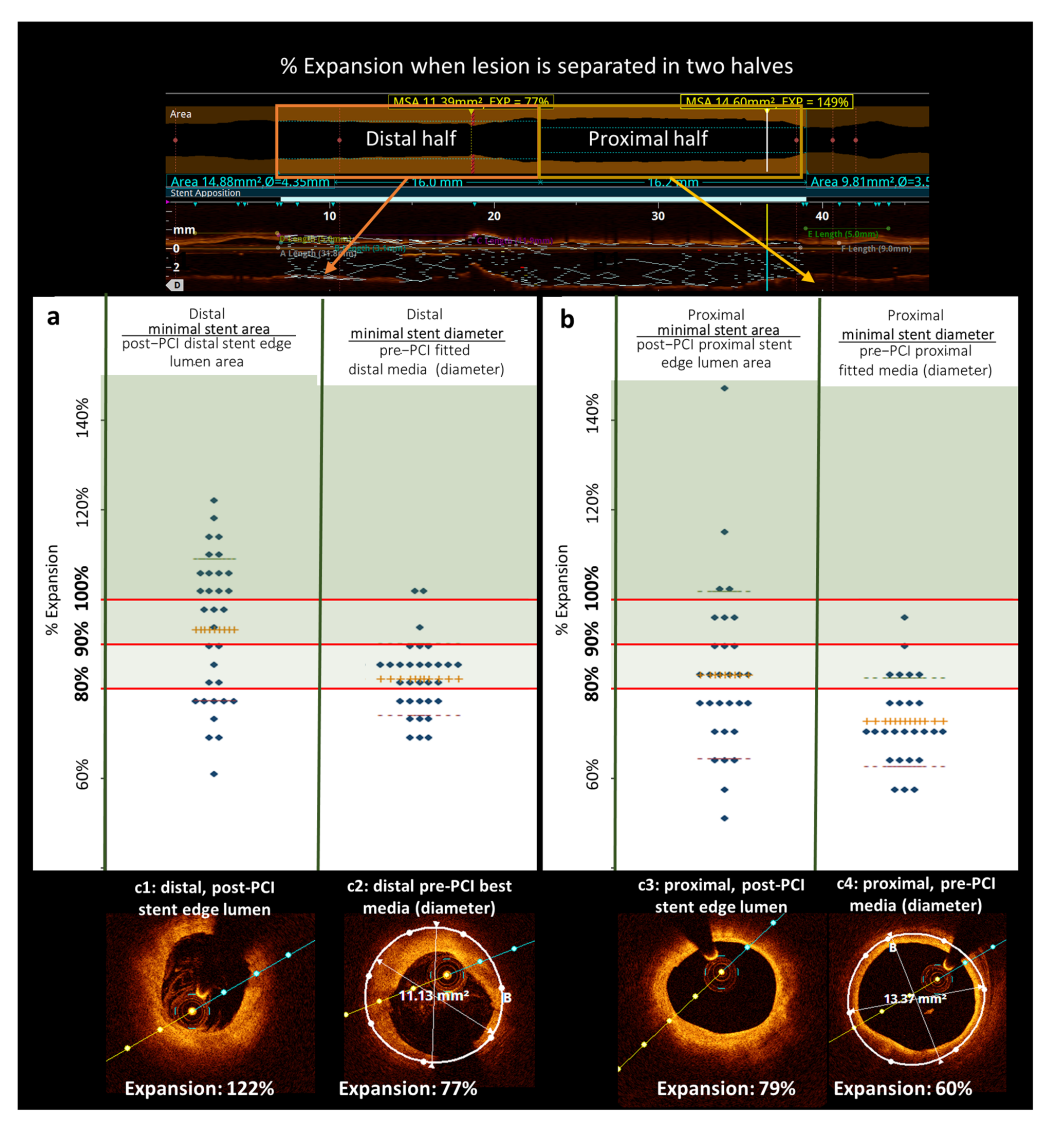
Shows dot-plots of expansion in % in a two-segment model, divided in the stent midline, and with the following reference definitions: (1) post-PCI stent edge lumen area (Method 6), or (2) pre-PCI best media contour within 0-10 mm in diameters (Method 4). When evaluating only the distal segment (Fig. 4**a**) and applying a cut-off value of 90%, final result of 18/32 cases were “acceptable” when post-PCI stent edge lumen area method was used, compared with 3/32 cases when the reference was based on pre-PCI media measurements. Applying an 80% cut-off, a total of 23/32 cases and 20/32 cases had acceptable expansion using same two methods. Similar results were found for analysis of proximal segments (Fig. 4**b)**

### Post-PCI evaluation of stent expansion. Segmental evaluation with step down

A SB with diameter ≥ 2.0 mm in the lesion segment was found in 14/32 (43.8%) cases. Obtaining two values for MSA (one per segment) when the stented segment was divided by the SB was feasible in 12/14 cases. Comparing these values with values when the same lesion segment was divided into two equally long segments changed distal MSA to be larger in 2 of 12 cases (6.4mm^2^ vs. 7.2mm^2^ and 6.0mm^2^ vs. 7.8mm^2^), resulting in a larger expansion value (supplementary Fig. 2, grey and purple dots). The proximal MSA measurements were changed in 7/12 cases (MSA smaller in 6/7 cases; larger in 1/7 cases) also resulting in differences in expansion calculations (supplementary Fig. 2).

## Discussion

This study compared published reference size methods for intravascular imaging and their possible effects on stent size selection and evaluation of stent expansion. We found that different reference methods lead to (1) major differences in estimated reference size, (2) possible differences in selection in stent size, and (3) major differences in evaluation and classification of post PCI stent expansion.

### Pre-PCI planning of the procedure – selection of stent - and balloon sizes

A main difference in reference methods was seen when 31 lumen-based estimations were compared to media-layer derived estimations ranging from mean 2.91 mm to mean 4.04 mm, respectively (Table 3).

The majority of identified studies recommended using a distal reference estimation for stent sizing. We found that media-based methods would result in selection of stents median 0.5 mm larger than lumen-based reference methods. Choosing the mean of the proximal and distal reference the stent selection was on average 0.75 mm larger based on media compared to lumen-based methods. Lumen size estimations neglect the regular age-related changes in vessel morphology [28]. Such frequent changes in the reference segments includes intimal thickening, positive vessel remodeling and formation of calcific, and lipid rich plaques [5]. Using angiographic visual estimation provides limited information about potential plaque burden in the reference segment or vessel wall remodeling [5]. A study by Mintz et al. showed that only 60% of 884 angiographically normal reference segments were normal when evaluated by IVUS [5]. In this regard, both angiographic visual estimation and imaging guided lumen based reference methods may frequently lead to undersized stent selection and too small expansion goals. Some clinical studies mandated to select stent size based only on the largest lumen, without evaluation of the vessel morphology [18]. A large lumen by coronary angiography could still have plaque or be a post-stenotic lumen enlargement with positive vessel remodeling [29]. Incorrect reference size estimation of more than 0.5 mm could lead to suboptimal implantation result with potential clinical impact. A particular issue is the selection of a stent platform where the maximal achievable stent dimensions are not sufficient for optimal stent expansion.

Recent imaging-guided studies aimed to overcome the limitations with lumen-based reference sizing by measurements defined by the external elastic lamina (EEL). Defining the media layer and not specifically the media-adventitia transition (EEL) was selected in this study due to a reasonable higher in-procedure feasibility and a limited error between the two definitions estimated to 0.10-0.15 mm. IVUS and OCT are in many ways similar in their capacity to define the different vessel layers, plaque types and stent dimensions. However, several key differences do exist [30]; with an image resolution of 10 μm, OCT has 10 times greater resolution compared with IVUS (20-40 μm), while the tissue penetration by OCT is limited (OCT: 1-2 mm, IVUS: 3-8 mm), in particular in presence of lipid plaques [31]. Due to the high attenuation of lipid limiting the media detection behind lipid plaque it was proposed that EEL/media estimations are only feasible using IVUS compared with OCT [10]. The ILUMIEN III study found that physicians were able to identify EEL > 180 degrees in-procedure using OCT in 84% of cases. The EEL was detected in 95% of OCT cases during subsequent corelab analysis. The higher spatial resolution of OCT in combination with required blood clearance offers a clear interface between healthy-looking segments and non-healthy looking segments [32]. The in-procedure feasibility of the individual reference methods may vary according to presence of thrombus or the level and composition of plaque in the reference segment. Such mechanisms should be clarified in future clinical evaluation of in-procedure reference size estimation.

### Post PCI evaluation of stent implantation – evaluation of stent expansion

Multiple definitions of “optimal stent expansion” have been proposed (Table 2). Stent underexpansion is an important known risk factor for stent failure. Hence, optimal and feasible evaluation of stent expansion is therefore of particular importance [6]. Expansion is assessed using either an absolute in-stent minimal stent area or diameter, or as a relative value expressed as minimal stent area/diameter to a reference size estimate.

Although expansion criteria by absolute values have been applied in several studies [15, 26, 33, 34], the strategy is infeasible when treating small vessels (< 2.5 mm) and may allow for accepting stents with expansion substantially below the reference size in large vessels. The NOBLE IVUS substudy indicated that relative rather than absolute expansion values predict prognosis in large vessels including the left main coronary artery [35]. The present study showed that evaluation of expansion as a relative value depends on the selected reference size estimation method. This includes which reference/references to use for calculation: (1) the distal or the mean of the distal and the proximal references, (2) lumen or media/EEL reference estimation or (3) area or diameter reference estimations, but also if one segment or divided segments should be used. Even with an optimal reference size, the threshold for optimal expansion is not firmly established with 80% and 90% expansion limits applied in investigated studies.

### Expansion by pre- or post-PCI reference estimations

Two ongoing large-scale randomized control trials (ILUMIEN IV Trial [27] and the OCTOBER Trial [9]) are both evaluating routine OCT guidance for stenting complex coronary lesions. The studies apply different approaches for evaluation of stent expansion. Stent expansion in the ILUMIEN IV trial is defined by MSA to the post PCI lumen area. In the OCTOBER trial expansion is evaluated by MSA to the pre-PCI reference estimation. This study showed that such very important difference in definitions can result in difference in post-PCI evaluation of expansion ranging from 100 to 64% in the same case. Selecting a reference vessel segment is not trivial. The vessel lumen immediate next to the post-PCI stent could be influenced by stent overexpansion, geographical miss, plaque shift or coronary artery dissection [36, 37]. In this study ≈ 40% of cases showed the largest post-PCI OCT lumen just at the stent boarder, indicating that the edge segment closest to the stent was influenced by the stent-implantation. Using a reference measured as post-PCI stent edge lumen for evaluation of stent expansion therefore may result in a larger reference-value and a smaller expansion in comparison to the corresponding pre-PCI lumen. Numerous trials reviewed in this study did not state whether pre- or post-reference estimations were used for expansion evaluation [17, 19, 23, 24, 36, 38].

### Expansion – area or diameter

Most studies evaluate the expansion in relation to area-estimations. However, the clinical feasibility in calculating % of an area seems offhand limited and inconvenient when compared to % of diameter. Since percentages of an area cannot be transferred directly to percentages of diameter (e.g. $${0.90 x\left(\frac{d}{2}\right)}^{2}x \pi \ne$$$${\left(\frac{0.9 x d}{2}\right)}^{2}x \pi$$), physicians must be careful not to transfer cut-off values based on an area-protocol to a diameter-protocol.

Our findings indicate that reference methods (1) should be vessel and not lumen-based, (2) should be applicable to long lesions with major diameter shifts, (3) should identify reference segments more than 2 mm from the stent edge, and (4) potentially be based on diameters to provide more actionable measurements for selecting balloons sizes. The post hoc nature and limited number of cases in this study do not allow for firm conclusions. It is important to establish a future consensus based on clinical outcome data expected from ongoing randomized trials.

### Limitations

Retrospective analyses are inherently limited by the fact that included data was collected for a different purpose. Measurements were performed in a core laboratory setting and not during angiographic procedures. Thus, it is unknown if the applied corelab analysis resemble measurements performed quickly during a procedure. The theoretic choice of stent size was performed according to established criteria but could differ from decisions made by treating physicians. Matching of pre - and post -PCI OCT-scans is limited by cardiac motion artifacts known to occur in 20–30% of cases [39]. To reduce this inherent limitation, visual confirmation of matched cross sections was performed by an experienced OCT observer.

## Conclusion

Differences in published methods for in-procedure analysis of intravascular imaging leads to major and concerning differences in coronary reference size, stent diameter selection, and in evaluation of stent expansion.

### Electronic supplementary material

Below is the link to the electronic supplementary material.

Supplementary Material 1
