# Supplementary material for: Comparison of definitions of coronary artery reference sizes and effects on stent selection and evaluation of stent expansion
Source: Int J Cardiovasc Imaging. 2023 Jul 5;39(9):1825–37. doi: 10.1007/s10554-023-02890-2 (PMC10520108; doi:10.1007/s10554-023-02890-2)
Supplement: Supplementary file 1 — Supplementary Material 1 [file 10554_2023_2890_MOESM1_ESM.docx]

**Supplementary - Table 1,** Overview of randomized control trials using DES and imaging-guidance

| HOME DES IVUS | 2010 | 210 patients with complex lesions | IVUS (poct-PCI) vs. angiographic guidance |
| --- | --- | --- | --- |
| Habara et al | 2013 | 70 de novo lesions | IVUS vs. OCT guidance |
| The AVIO Trial | 2013 | 284 complex lesions | IVUS vs. Angiographic guidance |
| CTO-IVUS | 2015 | 402 patients with CTO | IVUS vs. Angiographic guidance |
| AIR-CTO | 2015 | 230 patients with CTO | IVUS vs. Angiographic guidance |
| IVUS XPL | 2015 | 1440 long coronary lesions | IVUS vs. Angiographic guidance |
| OCTACS | 2015 | 100 NSTEMI | OCT (poct-PCI) vs. Angiographic guidance |
| RESET | 2015 | 543 patints with long lesions | IVUS vs. Angiographic guidance |
| Tan Q et al. | 2015 | 123 ULMCA - elderly patients | IVUS vs. Angiographic guidance |
| Zhang et al. | 2016 | 84 small vessels | IVUS vs. Angiographic guidance |
| ILUMIEN III | 2016 | 450 native lesions | IVUC vs. OCT vs. Angiographic guidance |
| DOCTORS | 2016 | 240 patients NSTEMI | OCT vs. Angiographic guidance |
| OCT STEMI | 2017 | 201 patients with STEMI | OCT vs. Angiographic guidance |
| OPINION | 2017 | 829 patients, simple lesions | OCT (OFDI) vs. IVUS |
| ULTIMATE | 2018 | 1448 all-comer patients | IVUS vs. Angiographic guidance |
| Illumien IV | Ongoing | Upto 3656 high risk patients | OCT vs. Angiographic guidance |
| OCTOBER | Ongoing | 1200 patients with bifurcations | OCT vs. Angiographic guidance |

**Supplementary - Table 2**: Reference description or definition

| Reference definition | NA | Lumen area adjacent to target +  most normal appearance +  free of lipidic plaque (pre-PCI) | Lumen area adjacent to target +  < 40% plaque burden | Largest lumen area (distal or proximal) with plaque burden < 50%  (within the same segment) | Largest lumen (distal or proximal)  with the least plaque  (within the same segment) | Most normal looking (post-PCI) no specific position | Lumen/vessel area (pre-PCI, non-specifik position) | Reference lumen area 5mm margin proximal/distal to stent (post PCI) | Mean of prox and distal lumen or vessel 1-5mm on either side of the stented segment (post-PCI) (MUSIC criteria)(1) | Media-to-media diameter/area or  EEL-to-EEL diameter/area | Lumen diameter if health looking with no intimal thickening | Mean proximal + distal EEL | Mean prox + distal vessel lumen  (most "normal-looking" CSA) |
| --- | --- | --- | --- | --- | --- | --- | --- | --- | --- | --- | --- | --- | --- |
| HOME DES IVUS |  |  |  |  |  |  |  | x  (IVUS) |  |  |  |  |  |
| Habara et. al. |  |  |  | X  (OCT) |  |  |  |  |  |  |  |  |  |
| The AVIO Trial |  |  |  |  |  |  |  |  |  | x  (IVUS) |  |  |  |
| CTO-IVUS |  |  |  |  |  |  | x  (IVUS) |  |  |  |  |  |  |
| AIR-CTO, |  |  |  |  |  |  | X  (IVUS) |  |  |  |  |  |  |
| IVUS XPL |  |  |  |  |  |  | x  (IVUS) |  |  | x  (IVUS) |  |  |  |
| OCT  ACS |  |  |  |  |  |  |  |  | X  (OCT) |  |  |  |  |
| RECET, |  |  |  |  | x  (IVUS  corelab) |  |  |  |  |  |  |  |  |
| OCT STEMI |  |  |  |  |  | X  (OCT) |  |  |  |  |  |  | x  (OCT) |
| Tan Q et al. |  |  |  |  |  |  | x  (IVUS) |  |  |  |  |  |  |
| Zhang et al. | x |  |  |  |  |  |  |  |  |  |  |  |  |
| Ilumien III |  |  |  |  |  |  |  |  |  |  |  | X  (OCT) |  |
| DOC  TORS |  |  |  |  |  |  | x  (OCT) |  |  |  |  |  |  |
| OPIN  ION |  | X  (OFDI) |  | X (IVUS) |  |  |  |  |  |  |  |  |  |
| ULTI  MATE |  |  | x  (IVUS) |  |  |  |  |  |  |  |  |  |  |
| Illumien IV |  |  |  |  |  |  |  | X  (OCT) |  | X  (OCT) |  |  |  |
| OCT  OBER |  |  |  |  |  |  |  |  |  | X  (OCT) | X (OCT) |  |  |

**Supplementary – Figure 1**: Bland-altman plot for selected post-PCI references


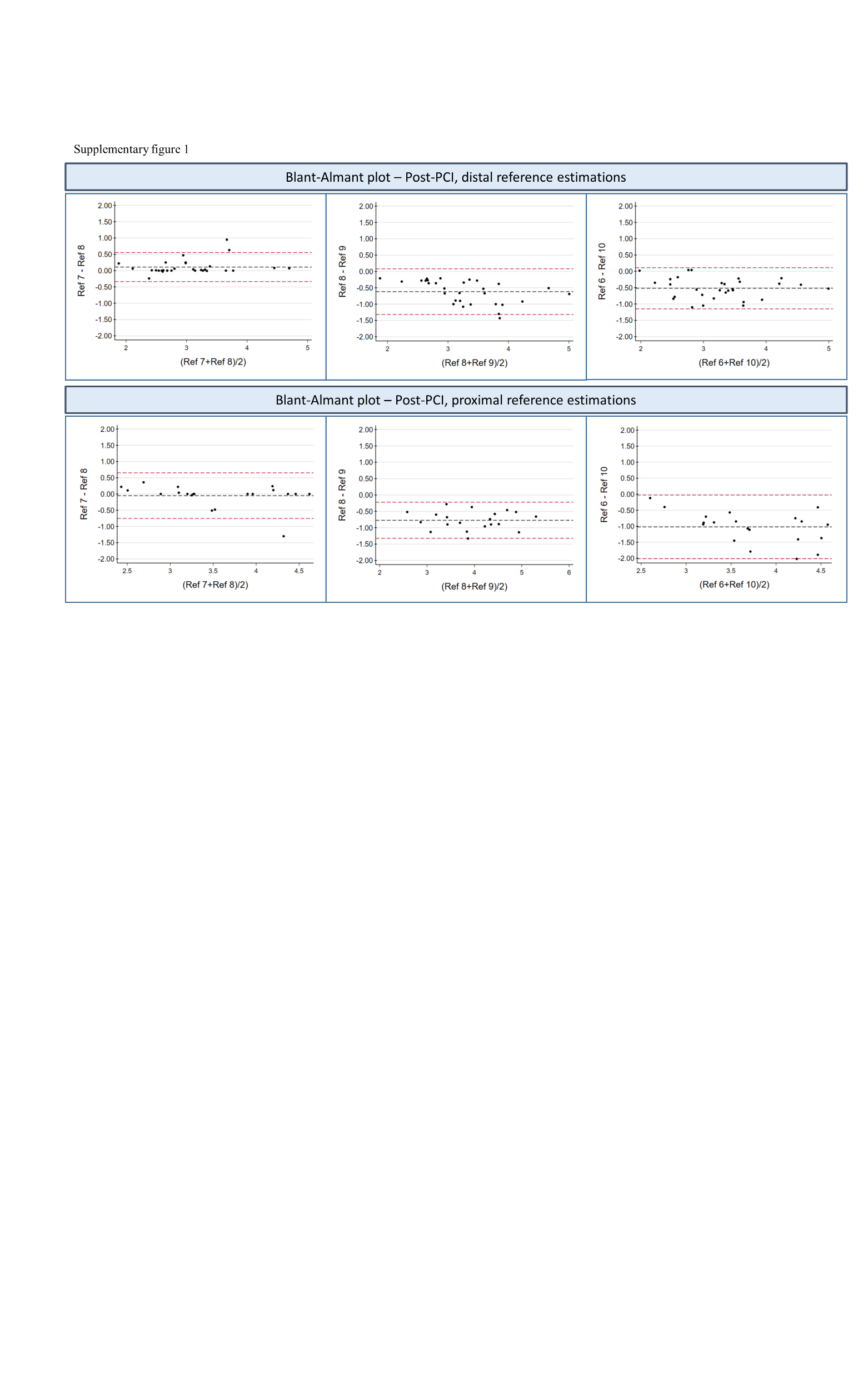


**Supplementary – Figure 2**: Expansion result from 12 cases with a side branch > 2mm in stented segment.


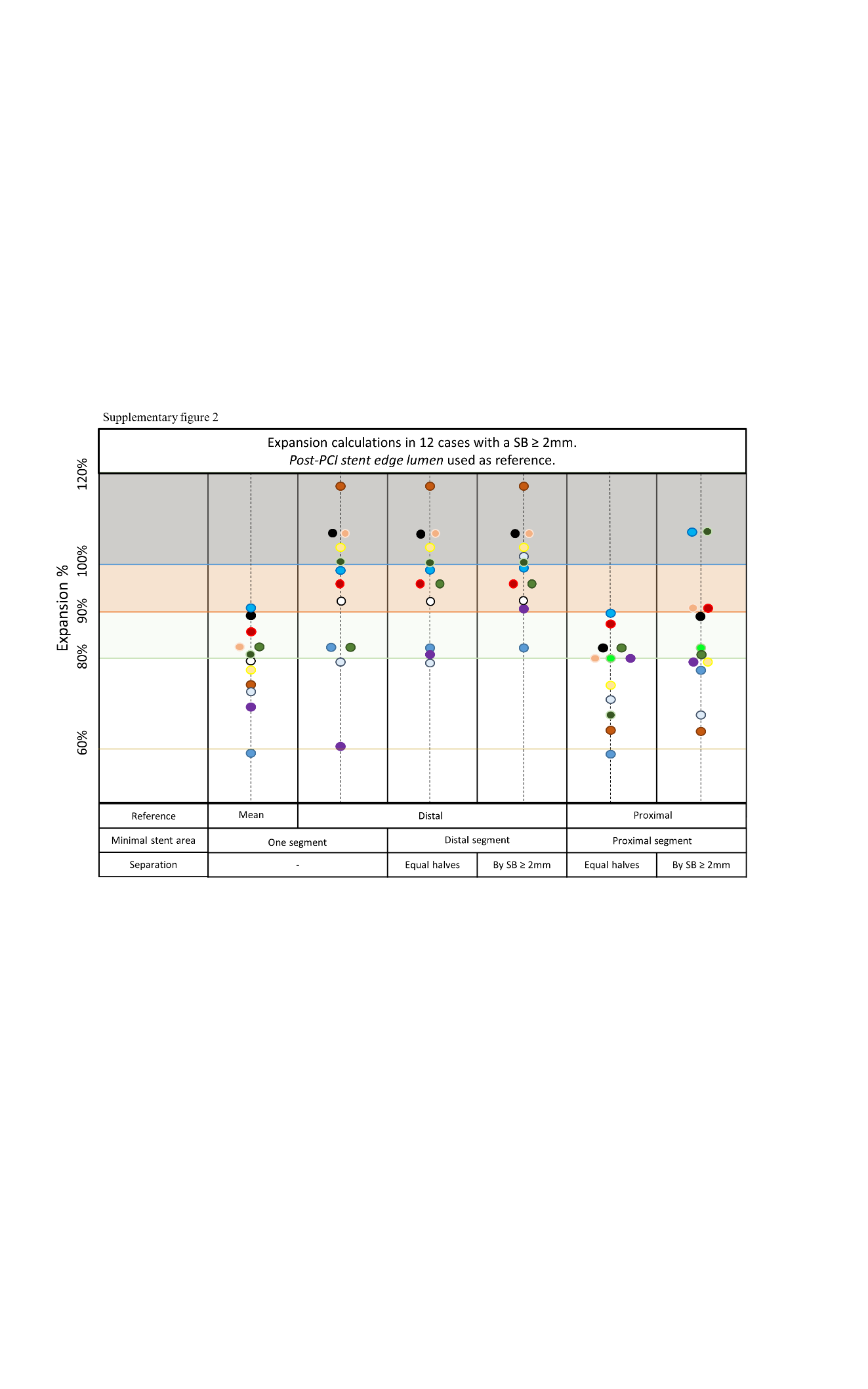


Results are based on post-PCI stent edge lumen reference areas and either 1) one segment MSA to mean reference (prox + distal), 2) one segment MSA to distal reference only, 3) MSA of distal half- stented segment to distal reference, 4) MSA of distal segment by SB > 2mm, 5) MSA of proximal half-stented segment to proximal reference, 6) proximal MSA of proximal segment separated by SB > 2mm. Green line indicates expansion=80%, orange line indicates expansion=90%, blue line indicates expansion=100%. *MSA: Minimal Stent Area*
